# Radiological, pathological and DNA remission in recurrent metastatic nasopharyngeal carcinoma

**DOI:** 10.1186/1471-2407-6-259

**Published:** 2006-10-31

**Authors:** Stephen L Chan, Edwin P Hui, Sing F Leung, Anthony TC Chan, Brigette BY Ma

**Affiliations:** 1 Department of Clinical Oncology, Prince of Wales Hospital, The Chinese University of Hong Kong, 30-32 Ngan Shing Street, Shatin, Hong Kong SAR, China

## Abstract

**Background:**

Circulating plasma Epstein Barr Virus DNA (EBV-DNA) is a sensitive and specific marker of nasopharyngeal carcinoma (NPC). The mainstay of treatment of metastatic NPC is systemic chemotherapy and resection for solitary metastasis. Despite high response rate to chemotherapy, complete remission is uncommonly seen.

**Case Presentation:**

We report a case of recurrent metastatic NPC in a 43-year-old man, who achieved complete remission three times with chemotherapy and surgery. Serial plasma EBV-DNA levels were measured during the course of disease. The patient had three episodes of recurrences of NPC manifested as distant metastasis. Both time, rise in the plasma EBV-DNA level preceded detection of recurrences by imaging. Following systemic chemotherapy, he achieved complete remission each time, of which was confirmed by 18-flourodeoxyglucose positron emission tomography and hepatectomy pathology. The plasma EBV-DNA level dropped to zero copy/ml at the time of each remission.

**Conclusion:**

This case highlights the high chemosensitivity of NPC by illustrating a rare occurrence of complete response of metastatic NPC to chemotherapy. This case also underscores the usefulness of EBV-DNA as a useful tool in monitoring NPC by its ability to detect early recurrence and excellent correlation with treatment response.

## Background

The recent development in nucleic acids in serum and plasma has opened up many new areas in molecular diagnosis. In the field of oncology, circulating plasma/serum Epstein Barr Virus (EBV) DNA in nasopharyngeal carcinoma (NPC) is a good example. The real-time polymerase chain reaction (RT-PCR) is a reliable and reproducible technique, which allows a rapid quantification of plasma EBV DNA, thus rendering it an attractive tool in the monitoring of treatment response [1,2]. The test is highly sensitive because it targets the BamHI-W repeat region of the EBV genome [2]. This test became available for clinical use at our oncology centre in 2002. Though it is uncommon to see patients with metastatic NPC achieve partial response to chemotherapy, it is very rare to observe complete response. Here we report a case which illustrates rare occurrence of complete response to chemotherapy and highlights good correlation between plasma EBV DNA and FDG-PET scan and pathology. Written consent was obtained from the patient for publication of this report.

## Case Presentation

A 43-year old man was referred to our cancer center in October, 2001 with stage IIb undifferentiated nasopharyngeal carcinoma (T2N1M0). He completed radical radiotherapy in December, 2001, and achieved complete remission afterwards. Although baseline plasma EBV DNA was not available, patient had undetectable plasma EBV DNA which was measured within one week after radiotherapy.

**Figure 1:**
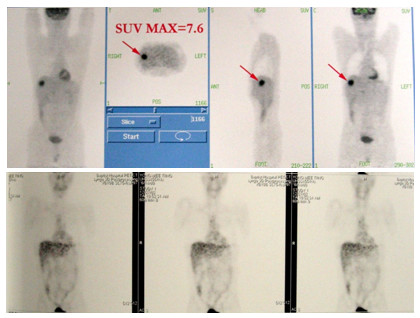
A solitary hepatic metastasis (SUV 7.6) was demonstrated by FDG-PET scan (above). After 6 courses of paclitaxel and carboplatin chemotherapy, follow-up scan showed there was resolution of the liver metastasis (below).

Upon a routine follow-up in August, 2002, the patient developed with an asymptomatic rise in the plasma EBV DNA level at 129,437 copies/ml. A subsequent FDG-positron emission and computered tomography fusion scan (FDG-PET-CT) showed a 4 cm hypermetabolic solitary metastasis in the right lobe of liver with a FDG standard uptake value (SUV) of 7.6 (Figure 1). Following treatment with six courses of paclitaxel and carboplatin chemotherapy, his plasma EBV DNA level fell to undetectable level, and a subsequent PET-CT performed immediately after completion of chemotherapy confirmed a reduction in the size and SUV of the liver metastasis (Figure 1). A right hepatectomy was performed at two weeks after the end of chemotherapy, and pathological examination of the resected specimen showed necrotic tissues without any evidence of viable malignant cells.

**Figure 2:**
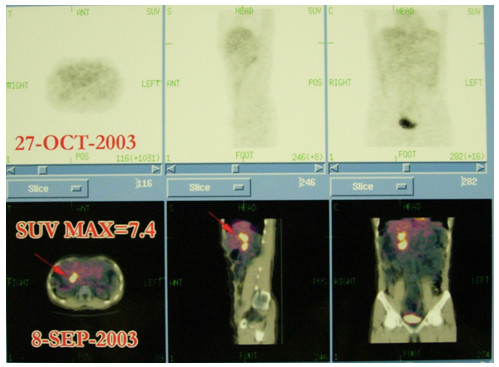
Another set of FDG-PET scan showing complete remission following 6 courses of gemcitabine and carboplatin chemotherapy: hepatic and pancreatic metastases disappeared in Oct, 03 after chemotherapy (above), compared with hypermetabolic lesions in September, 03 (below).

The patient enjoyed a disease-free period of about nine months until a second disease relapse in September, 2003, when he was found to have asymptomatic rise in plasma EBV-DNA level to 678 copies/ml. A subsequent PET-CT confirmed multiple hyper-metabolic recurrences in the liver, lung and pancreatic head (Figure 2). After 2 courses of second-line chemotherapy with gemcitabine and carboplatin, his plasma EBV DNA level fell to undetectable level which coincided with complete disappearance of metastatic lesions (i.e. complete remission) on PET-CT in October, 03 (Figure 2). He completed a total of 4 cycles of gemcitabine and carboplatin and remained disease-free until 4 months later in February 2004, when he developed a third relapse which was heralded by an asymptomatic and progressive rise in plasma EBV-DNA levels from 56 to 329 copies/ml over a 2 month period. A PET-CT performed in April 2004 confirmed recurrence in the liver and pancreatic head. He was re-treated with 6 cycles of gemcitabine and carboplatin chemotherapy, and again, his plasma EBV-DNA level became undetectable and PET/CT showed complete remission. Upon his last follow-up in November 2004, he remained disease-free for 7 months following the last chemotherapy treatment in 2004.

## Conclusion

**Figure 3:**
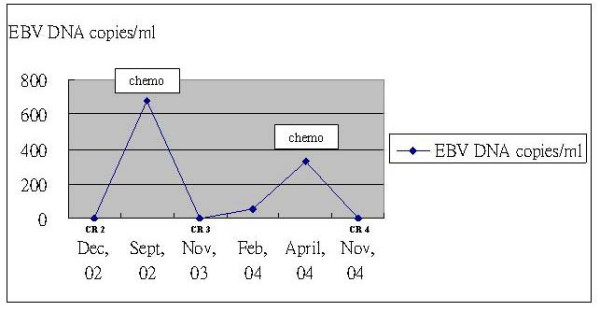
Each complete remission (CR) coincided with dropping of EBV-DNA level to zero copy/ml, and recurrence of disease was associated with rise in plasma EBV-DNA level.

In our patient, rising levels of plasma EBV DNA was consistently associated with evidence of distant recurrence as indicated objectively on PET-CT imaging (Figure 3 & Table 1), before the development of any clinical signs or symptoms of relapse. Second, radiological evidence of complete response (delineated by FDG-PET) to chemotherapy also coincided consistently with undetectable levels of plasma EBV DNA. We did not perform histological confirmation of PET-CT detected disease recurrence because the location and SUV intensity of those abnormalities were unlikely due to false-positive or inflammatory causes. Furthermore, studies have shown that PET is sensitive and specific in detecting recurrent nasopharyngeal carcinoma [3,4].

It is observed that patient subsequently developed recurrences though plasma EBV DNA dropped to zero copy/ml when he was having a relatively inactive disease (which is manifested as clinical and radiological remission). This is unlikely due to suboptimal EBV DNA assay because the assay used by our centre is well validated as reported previously [1,2,5]. It is noteworthy that although plasma EBV DNA is a sensitive tool in monitoring disease and tumor burden, zero copy/ml of EBV DNA does not necessarily equate complete eradication of disease, and close monitoring of clinical condition and EBV DNA level are still required. The case study also points to the importance of a well standardized and highly sensitive PCR method, because the relatively low level of EBV DNA detected in second and third relapse might be missed with a less sensitive PCR method [1,10].

Cell-free EBV DNA was first demonstrated in circulation of NPC patient by PCR amplification in 1998 [6]. Dependent on number of PCR cycles, modalities of PCR and various preparations of specimen, different assays of various sensitivity and specificity were subsequently developed for detection of circulating EBV DNA [1,7-10]. Several characteristics have enabled circulating EBV DNA to be widely used in clinical practice for management of NPC. In our institute, with the use of real time PCR, EBV DNA in plasma had been demonstrated to be a both sensitive (96%) and specific (93%) marker of NPC [1]. EBV DNA is undetectable in healthy individuals and those disease-free patients with history of NPC, while it is raised in plasma of most patients with active disease. Also, plasma EBV DNA has a role in staging and prognostication [11,12]: higher level of EBV DNA is detected in patient with more advanced UICC stage, and those patients with non-metastatic NPC have poorer prognosis when there is persistent high EBV DNA level after radical radiotherapy to nasopharynx. More importantly, detection of EBV DNA can be used for monitoring of NPC patients for any recurrence or metastasis [2,13]. It had been reported by that rise in EBV DNA might predate clinical relapse up to few months hence rendering earlier work-up and treatment feasible [13]. Other potential use of circulating EBV DNA remains to be elucidated. For example, the use of EBV DNA in screening general population for NPC is still under active research. There are also ongoing studies addressing the improved accuracy of detecting NPC with combined use of circulating EBV DNA and other serological markers. In our centre, we have already completed a prospective study looking into correlation between plasma EBV DNA and PET-CT imaging before and after radiotherapy in patients with advanced NPC. With this case, apart from reporting an uncommon occurrence of chemotherapy induced complete remission of metastatic nasopharyngeal carcinoma, we hope this case can highlight the good correlation between circulating EBV DNA and FDG-PET scan in disease monitoring of nasopharyngeal carcinoma.

## Competing interests

The author(s) declare that they have no competing interests.

## Authors' contributions

SLC Patient care, Concept & design, Preparation of manuscript

EPH Patient care, Critical revision

SFL Patient care, Critical revision

ATCC Patient care, Critical revision

BBYM Patient Care, Concept & design, Final revision

All authors read and approved the final manuscript

## Pre-publication history

The pre-publication history for this paper can be accessed here:



## Figures and Tables

**Table 1:** the correlation between plasma EBV-DNA level and clinical status; clinical and radiological remission is associated with falling level of EBV-DNA level to zero copy/ml.

**Time**	**EBV DNA copies/ml**	**Clinical Status**
Aug, 02	129837	1^st ^relapse
Dec, 02	0	2^nd ^remission
Sept, 02	678	2^nd ^relapse
Nov, 03	0	3^rd ^remission
Feb, 04	56	3^rd ^relapse
April, 04	329	
Nov, 04	0	4^th ^remission
